# Altered Ramadan fasting glycemic profiles of adults with type 1 diabetes reveal strong evidence of underestimated insulin adjustments: a 3-year observational study in Arab settings

**DOI:** 10.3389/fendo.2025.1399990

**Published:** 2025-05-29

**Authors:** Abdullah M. Alguwaihes, Ebtihal Y. Alyusuf, Areej Alrajeh, Metib Alotaibi, Mohammed E. Al-Sofiani

**Affiliations:** ^1^ Division of Endocrinology, Department of Internal Medicine, College of Medicine, King Saud University, Riyadh, Saudi Arabia; ^2^ Dallah Hospital, Diabetes Center, Riyadh, Saudi Arabia; ^3^ Division of Endocrinology, Department of Internal Medicine, Salmaniya Medical Complex, Manama, Bahrain; ^4^ Department of Internal Medicine, King Saud University Medical City, King Saud University, Riyadh, Saudi Arabia; ^5^ University Diabetes Center, King Saud University Medical City, Riyadh, Saudi Arabia; ^6^ Division of Endocrinology, Diabetes & Metabolism, The Johns Hopkins University, Baltimore, MD, United States

**Keywords:** Ramadan, intermittent fasting, type 1 diabetes, Saudi Arabia, flash glucose monitoring, continuous glucose monitoring

## Abstract

**Background:**

Adults with type 1 diabetes (T1D) who fast during Ramadan remain a severely understudied population in terms of changes in glycemic control, making evidence-based recommendations for insulin adjustments difficult in this age-group. To fill this gap, we aimed to prospectively observe the changes in glycemic control of young adults with T1D who fast during Ramadan.

**Methods:**

In this 3-year prospective study, we enrolled participants with T1D with flash glucose monitoring (FGM) data during the Ramadan periods of 2020-2022. CGM data for 4 weeks before, during, and after Ramadan were collected and analyzed. A sub-cohort of age-matched non-DM participants (N=49) who fasted during the Ramadan of 2022 were included for comparison.

**Results:**

A total of 76 participants were enrolled, of whom only 39 (19 males and 20 females, mean age 28.1 ± 8.4 years) completed the three-year follow-up. The mean duration of diabetes among these participants was 11.5 ± 8.9 years. Ten (26%) patients were on insulin pump, and 22 (56%) patients received Ramadan-focused education at baseline. Pooled glycemic trends during Ramadan showed two main abnormal glucose spikes: after Iftar (between 16:00-18:00 and 18:00-20:00), with a difference of 15.5mg/dL, and after Suhoor (between 0:00-2:00h to 4:00-6:00), with a difference of 18.8mg/dL. These abnormal glycemic indices persisted a month after Ramadan. In parallel, these glucose spikes were also observed in non-DM participants, but remained within normal limits.

**Conclusions:**

Ramadan fasting among adults with T1D in SA is associated with deterioration in glycemic control, with the highest glucose spikes observed after Iftar and Suhoor. These hyperglycemic episodes were most prominent during Ramadan and persisted for at least a month after. The present real-time evidence warrants the need to review insulin adjustments in this understudied group, focusing on high risk patients with T1D, including those with history of overindulgent behavior during Ramadan.

## 1 Introduction

Ramadan is a holy Islamic month during which Muslims observe fasting from dusk until dawn and abstain from food or drink (1). Suhoor, the pre-dawn meal, is the meal consumed before fasting begins, while Iftar is the meal that marks the moment to break the fast at sunset. While intermittent fasting has been shown to offer several benefits (2, 3), it differs from Ramadan fasting in terms of flexibility of fasting days, the latter being stricter as it has to be implemented for a month. Ramadan in Saudi Arabia (SA) is associated with extravagant changes in lifestyle and eating habits; people tend to consume larger portions, including higher consumption of traditional sweets and sweetened beverages which are culturally driven (4–6). Furthermore, sleeping patterns are reversed during Ramadan in SA, resulting in lower physical activity and higher levels of evening cortisol, leading to a paradoxical increase in insulin resistance (7, 8).

For patients with type 1 diabetes (T1D) who choose to fast during Ramadan, as they need to re-adjust insulin doses and timing to minimize the risk of abnormal glycemic changes (9). Moreover, because of the long duration of absolute fasting particularly during summer, additional risks should be considered in patients with T1D, including dehydration, hypoglycemia, and possibly an increased risk of diabetic ketoacidosis (10–12). Despite medical and religious exemptions, a considerable number of T1D patients insist on fasting (1, 13). An epidemiological study across several Muslim countries estimated that 42.8% of Muslims with T1D fast for ≥ 15 days and this was highest in SA at 71.6% (12). A recent survey that included T1D patients and conducted during the coronavirus pandemic found that 71.1% of respondents intended to fast (14). However, only 26.8% managed to fast for the full month, while 45% fasted for more than 21 days. Additionally, 60.7% of participants reported hypoglycemia episodes (14). Several guidelines recommended against fasting during Ramadan for high-risk T1D patients due to lack of available evidence (9, 10, 15). Nevertheless, some studies have shown that it can be safely accomplished in selected patients (16, 17).

Novel technologies seem to provide promising benefits for T1D patients fasting during Ramadan (9, 15). Real-time continuous glucose monitoring (RT-CGM) and flash glucose monitoring systems (FGM) provide comprehensive evaluation of glucose measurements and variability, enabling patients and healthcare professionals to make better-informed decisions (18–23). Additionally, insulin pumps may help individuals with T1D maintain better clinical outcomes (13, 24). Despite the high prevalence of fasting during Ramadan among patients with T1D, there remains a significant gap in the understanding of the impact of this type of fasting on glycemic control, notably among adults with T1D. To fill this gap, we examined the changes in glycemic control among adult patients with T1D who attempted to fast before, during, and after Ramadan and how these changes may have differed from the glycemic changes in non-T1D individuals.

## 2 Methods

### 2.1 Study design and participants

**Figure 1:**
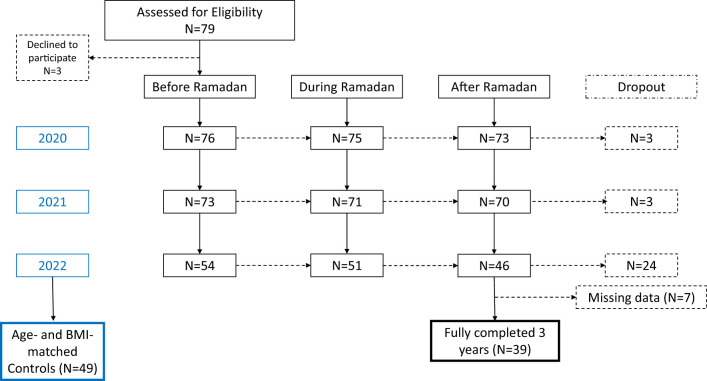
Flowchart of participants.

This is a real-world observational study that initially included 79 outpatients with T1D who had FGM data during 4 weeks pre-Ramadan, 4 weeks during Ramadan, and 4 weeks post-Ramadan during 2020, 2021 and 2022, at the diabetes clinics of King Saud University Medical City (KSUMC), Riyadh, Saudi Arabia. The inclusion criteria for the study were patients with T1D, aged ≥15 years, who intended to fast during one or more of the Ramadan periods of the three years of the study, and had available FGM data. We excluded patients with T2D, patients with T1D who didn’t attempt fasting during Ramadan or with no FGM data during Ramadan in any of the study periods. Moreover, 49 healthy individuals without diabetes were included in Ramadan 2022. The participants without diabetes were recruited from hospital staff and patients’ companions who consented to wear the sensor for Two weeks during Ramadan 2022 and share their data for the study. Demographic and clinical data such as age, gender, medications, comorbidities, duration of diabetes, HbA1c, and the status of receiving focused-Ramadan education were extracted from the electronic medical records for all participants with T1D. The total fasting hours per day during Ramadan can vary within a year and from year to year. However, the fasting hours during the three study years were around 14.5 hours per day starting at around 04:00 h and ending at around 18:30 h. By the end of the study, 39 participants (19 males and 20 females) remained and were included for analysis (see **Figure 1**). It is worthy to note that the sudden drop of participants in the last year (2022) was primarily due to the limited availability of sensors, and not necessarily due to dropouts. The study was approved by the institutional review board College of Medicine, King Saud University Riyadh, Saudi Arabia (Ref 21/0439/IRB).

### 2.2 Glycemic measures using flash glucose monitoring

All participants used the first generation Freestyle Libre 14-day System (Abbott Diabetes Care, Alameda, CA) which is a factory-calibrated flash glucose monitoring sensor with a mean absolute relative difference of 11.4% (25). In this study, we utilized CGM glycemic metrics with their widely accepted definitions including average sensor glucose, glucose management indicator (GMI), time in range (TIR) (i.e. glucose: 70–180 mg/dl), time above range level 1 (TAR-1) (i.e. glucose: 181–250 mg/dl), TAR level 2 (TAR-2) (i.e. glucose: >250 mg/dL), time below range level 1 (TBR-1) (i.e. glucose: 54–70 mg/dl), TBR level 2 (TBR-2) (i.e. glucose: <54 mg/dl), glycemic variability, as measured by the coefficient of variation (CV), sensor active time, and the number of daily scans, which were obtained from the Freestyle LibreView platform (26).

### 2.3 Assessment of glycemic outcomes

The following CGM metrics were evaluated in all the study participants at 4 weeks pre-, 4 weeks during, and 4 weeks post-Ramadan: Average sensor glucose, GMI, TIR, TAR-1, TAR-2, TBR-1, TBR-2, sensor active time, and the number of daily scans. Average sensor glucose throughout the day during each of the study periods, pre-, during, and post-Ramadan, were analyzed by looking at the patterns and trends of sensor average glucose changes during the day hours and postprandial hours. For controls (individuals without diabetes), similar CGM metrics were only reported during the month of Ramadan. For this group, sensor average glucose throughout the day during Ramadan of 2022 was analyzed by looking at the patterns and trends of sensor average glucose changes during the day hours and postprandial hours. Moreover, laboratory HbA1c within the normal range were required from this group at enrollment.

### 2.4 Statistical analysis

Analyses were conducted using SPSS software version 28.0 Descriptive statistics, such as mean ± SD for continuous variables and frequencies and percentages for categorical variables, were reported. Repeated measures analysis of variance (ANOVA) was used to compare differences over time. The estimated marginal means and estimated differences from the model were reported as the estimated mean with a 95% confidence interval (CI). All reported p-values are two-tailed, and p<0.05 was considered statistically significant.

## 3 Results

### 3.1 Baseline characteristics

The study included a total of 39 T1D participants out of 79 who started in 2020, with 20 (51%) of them being female. The mean age of the participants was 28.1 ± 8.4 years, and the mean duration of diabetes was 11.5 ± 8.9 years. Additionally, 10 (26%) participants were on insulin pump, and 22 (56%) had medical record documentation that they received Ramadan-focused education since 2020. Complete CGM data for all three years of the study was available for all participants. **Table 1** presents the baseline characteristics and number of participants with T1D. Moreover, the study included 49 age-and BMI-matched participants (34 females or 69%). The participants had a mean age of 27.4 ± 8.0 years. The mean BMI was 24.0 ± 4.5 kg/m^2^, and the mean HbA1c was 5.2 ± 0.3%.

**Table 1:** Characteristics of participants.

Parameter	T1D	Controls
N (M/F)	39	49
Male/Female	19/20	15/34
Age (years)	28.1 ± 8.4	27.4 ± 8.0
Type 1 Diabetes duration (years)	11.5 ± 8.9	–
Body mass index (kg/m^2^)	25.3 ± 4.4	24.0 ± 4.5
HbA1c (%)	8.7 ± 1.9	5.2 ± 0.3
Insulin Delivery Modality		
Pump	10 (26)	–
Multiple Daily Injections	29 (74)	–

Data presented as frequencies (%) and mean ± standard deviation.

### 3.2 Glycemic indices overtime in people with T1D

A comparison of glycemic metrics across the three years of the study period is shown in **Table 2**. Insulin doses received before and during Ramadan were highest in 2022 as compared to previous years (p-values 0.02 and 0.01, respectively). There were no significant differences observed over time in terms of Aspart use, daily and suggested insulin doses before and during Ramadan, ICR as well as pre- and post-BMI over time. In terms of glycemic indices, mean HbA1c was worst before Ramadan of 2020 (8.7 ± 1.9) for all participants, which was significantly higher than successive years (p=0.02). On the other hand, mean glucose variability was highest in 2022 as compared to previous years (p=0.01). The rest of the glycemic indices were comparable over time (**Table 2**).

**Table 2:** Changes in glycemic indices overtime.

	2020	2021	2022	P-value
N	39			
Received Ramadan Education	22 (56)	26 (67)	16 (41)	
Insulin dose before Ramadan	26.4 ± 12.4	25.1 ± 9.3	28.4 ± 10.3	0.02
Suggested Insulin dose in Ramadan	23.1 ± 12.3	23.0 ± 9.3	25.8 ± 8.7	0.01
Aspart dose before Ramadan	27.4 ± 10.0	30.4 ± 9.4	35.6 ± 17.0	0.16
Suggested Aspart dose in Ramadan	19.6 ± 6.5	19.4 ± 7.6	29.6 ± 9.1	0.16
Daily total insulin dose before Ramadan	55.3 ± 19.1	58.5 ± 17.0	70.7 ± 23.9	0.32
Suggested total insulin dose for Ramadan	41.6 ± 14.2	43.4 ± 17.7	62.4 ± 16.5	0.38
Pre-BMI (kg/m^2^)	25.3 ± 4.4	25.6 ± 4.3	26.6 ± 4.8	0.09
Post-BMI (kg/m^2^)	25.2 ± 4.5	25.5 ± 4.3	27.9 ± 4.6	0.33
HbA1c Before Ramadan	8.7 ± 1.9	7.8 ± 1.0	7.5 ± 0.9	0.02
Active Time Sensor	82.0 ± 21.0	81.9 ± 22.9	88.6 ± 12.1	0.08
Glucose (mg/dl)	167.2 ± 39.9	165.9 ± 30.8	166.9 ± 30.6	0.94
GMI	7.2 ± 0.8	7.2 ± 0.7	7.3 ± 0.7	0.89
GMI mmol	55.7 ± 8.9	55.7 ± 7.3	56.3 ± 7.9	0.92
Glucose Variability	37.9 ± 7.6	36.7 ± 6.5	39.2 ± 6.2	0.01
Daily Scans	10.7 ± 6.8	10.2 ± 7.3	10.8 ± 8.7	0.84

Data presented as mean ± standard deviation; p-value significant at <0.05.

**Figure 2:**
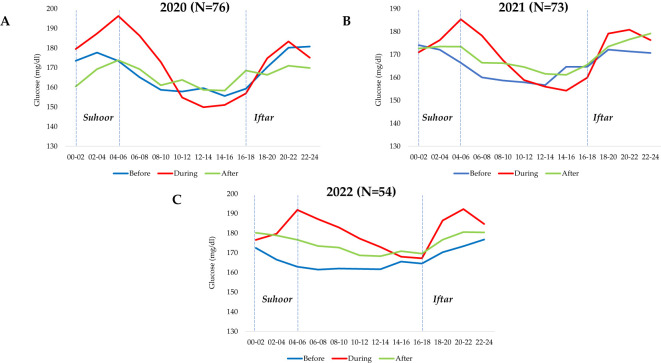
Average 24-hour glycemic pattern of T1D participants before, during and after Ramadan (2020-2022). **(A)** 2020. **(B)** 2021. **(C)** 2022.

**Figure 3:**
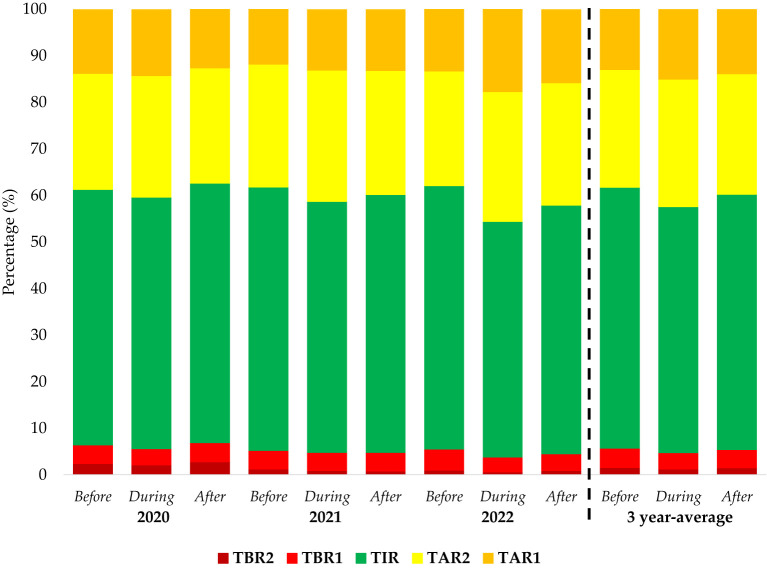
Time in ranges before, during and after Ramadan of the years 2020, 2021 and 2022.

**Figure 2** shows the mean glycemic pattern of T1D participants before, during and after Ramadan for each year while **Figure 3** shows the pooled data for 3 successive Ramadan. In both figures, 2 abnormal glucose spikes have been consistently observed overtime and in pooled analysis, all of which occurred during the period of Ramadan. In the pooled data (**Figure 3**) before Ramadan, the two-hour time bucket with the lowest average glucose level was observed at 10:00-12:00 h (159.2 ± 26.2), whereas the highest average glucose level was observed at 22:00-24:00 h [176.1 ± 27.4] (not mentioned in table). During Ramadan, the lowest average glucose level was observed at 14:00-16:00 h (157.8 mg/dL ± 25.8). The highest average glucose levels were observed after Iftar at 20:00-22:00 h (185.5 ± 27.6), and after Suhoor at 4:00-6:00 h (191.1 ± 27.1). Glycemic trends during Ramadan showed that the highest glucose spike occurred after Iftar, between 16:00-18:00 h and 18:00-20:00 h, with a difference value of 15.5 mg/dL and after Suhoor (between 0:00-2:00h to 4:00-6:00), with a difference of 18.8mg/dL (not shown tables). Glycemic values before, during and after Ramadan for each successive year are presented in **Supplementary Table S1**.

With respect to other CGM indices, no significant differences were seen in TAR1, TAR2, TIR, TBR1 and TBR2 before, during and after Ramadan of the years 2020 and 2021. In 2022 however, TAR1 and TAR2 were observed to be significantly higher during Ramadan than before and after (p-values 0.008 and 0.007, respectively). In parallel, TIR and TBR1 were also significantly lower during the Ramadan as compared to before and after (p-values 0.001 and <0.001, respectively), with TBR2 during Ramadan also being significantly lower only during before Ramadan of 2022 (p=0.02) (**Supplementary Table S2**). Pooled time indices showed no significant differences. Time in ranges over time were presented as **Figure 3**.

### 3.3 Impact of Ramadan fasting on glycemic indices in individuals without diabetes during Ramadan

**Figure 4:**
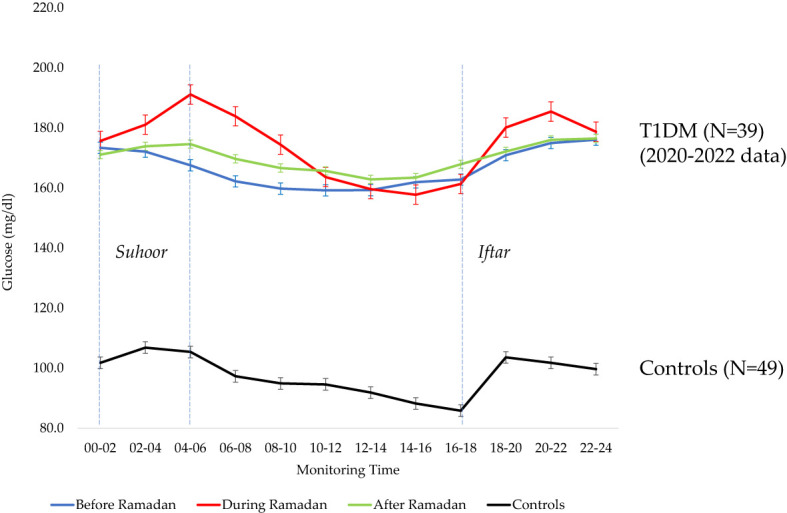
Pooled glycemic metrics of T1D participants before, during and after Ramadan versus Controls.

**Figure 4** shows the glycemic indices of control participants during the Ramadan of 2022. The lowest average glucose level was observed at 16:00-18:00 h (85.8 ± 7.8). The highest average glucose level after Iftar was observed at 18:00-20:00 h (103.6 ± 10.7), and the glucose change between the lowest and highest average glucose was 17.8 mg/dL (16.1 to 19.5) (**Supplementary Table S1**).

## 4 Discussion

In this real-world study, findings revealed a tendency for patients and/or their physicians to adopt an overprotective behavior to avoid hypoglycemia during Ramadan leading to an increase in time spent above the recommended glycemic range and a decrease in time spent within and below the target range. The pattern of glucose fluctuation (timing of the lowest and highest average glucose level) seen on FGM confirms the significant changes in lifestyle taking place during Ramadan in Saudi Arabia. The pattern of glucose levels during Ramadan, as observed in the present study, is consistent with previous studies (27–29). The average glucose levels slightly rise two hours before the Iftar meal, which could be attributed to the waning effect of the basal insulin dose and/or the increase in stress hormones due to prolonged fasting. Following the Iftar meal, there was a sharp increase in average glucose levels with a pronounced excursion that lasted for four hours, followed by a moderate decrease. The same pattern was observed after the Suhoor meal, with an increase in average glucose levels to the same extent and duration as the Iftar meal. During fasting, glucose levels gradually declined, reaching their lowest average at 14:00-16:00 h. This pattern was significantly different from that observed outside of Ramadan in several ways. First, the post-meal amplitude of glucose excursion during Ramadan was much higher. Second, there were higher fluctuations during Ramadan, and the difference between the lowest and highest average glucose was significantly greater. Third, contrary to the general assumption that TBR would be higher during Ramadan due to prolonged fasting (18), TBR was lower in this study in only the last year of the 3-year observation (2022). Consequently, GMI and TAR were all significantly higher during Ramadan than before and after of the same year.

The FGM data during Ramadan in healthy participants without T1D showed a similar pattern of glucose changes, albeit within normal, suggesting that the changes we are observing in patients with T1D is not only due to miscalculated/mismatched insulin requirement but also to the largely underestimated effect of cultural habits and diet taking place during Ramadan. As expected, not only the amplitude of postprandial hyperglycemia is greater in T1D patients, but also the duration of hyperglycemia which subjects patients with T1D to complications. The postprandial glucose excursion during Ramadan in healthy participants is similar to that reported in a previous study without Ramadan fasting (30). In patients with T1D, not only insulin management is important but also the effect of cultural diet on their glucose should be taken in consideration.

In the present study, the glucose trends during Ramadan exhibit the lowest average glucose levels between 14:00 to 16:00 h, which may be the time associated with a higher risk of hypoglycemia. Therefore, patients should be educated to frequently check their glucose levels during this time period, avoid strenuous physical activity, and respond immediately to any symptoms of hypoglycemia (27). It is highly important to bear in mind that patients living with T1D are prone to hypoglycemia unawareness. Previous research has found that patients with T1D spent an average of 1.39 hours per fasting day in hypoglycemia, with 8% of cases documented as severe yet asymptomatic (18).

The DAR-MENA T1D showed similar rates of confirmed and symptomatic hypoglycemia during Ramadan compared to before Ramadan (4). Other studies reported no increase in the time spent in hypoglycemia during Ramadan, but found no significant differences in terms of average glucose, GMI, or TAR (31–36). The present study found similar observations in the first 2 years of the study, with the final year showing significant differences in all time range indices. It is worthy to note that compared to years 2020 and 2021, most pandemic restrictions were lifted in Saudi Arabia from March 2022, a month before Ramadan, with all restrictions removed in June 2022. This complete removal of preventive measures that led to heightened social mobility, full opening of the fast food and dining industry, as well as outdoor recreational activities may have partially, but not fully explain, the differences observed in that year.

The present study results showed better glycemic control when compared to a previous study of glycemia during Ramadan in patients with T1D, which reported a TIR of 42%, TAR of 48%, and TBR of 10%. The study reached the same conclusion that there is a higher rate of hyperglycemia than hypoglycemia related to Ramadan fasting (27).

The unique addition of the current study is providing insight to what happens after Ramada. Although the values of the glycemic metrics improved during the month after Ramadan, they did not return to pre-Ramadan levels completely. Hence, fasting during Ramadan was associated with at least two months of disturbance in glycemic control, which highlights the need to reshape the conventional perception of Ramadan fasting as a risk factor for only hypoglycemia. More efforts and attention should made for the period after Ramadan not to prolong the duration spent in hyperglycemia.

The present study has some limitations. First, because this was a real-world study, we could not control or adjust dietary intake diversity among participants, which may have influenced our results. Second, we did not collect data on the timing and reasons for fast-breaking or hypoglycemic events, as well as insulin types used and day-to-day dose adjustments made. Finally, not all patients had complete data for the entire three-year period, but we reported estimated means and confidence intervals to illustrate the effect size of changes.

Despite these limitations, our study provides valuable insights into glucose profiles before, during, and after Ramadan fasting in adult patients with T1D using FGM. One of the strengths of this study is that it focused solely on patients with type 1 diabetes, young adults in particular, filling the needed data for an understudied population in T1D. Additionally, this study included patients’ data for three years, providing a more robust dataset.

The results of the current study have important clinical implications for patients with T1D and medical professionals alike since robust data for adults with T1D are scarce. Diabetes education and management strategies should be individualized and include diet and cultural habits for Ramadan, insulin dosing and timing tailored for Ramadan to help T1D patients safely fast, and counseling on after-Ramadan management.

## 5 Conclusions

This real-world observational study provided a unique and comprehensive look at the glycemic changes among young adults with T1D who attempted to fast before, during, and after Ramadan in SA, and revealed how Ramadan fasting can be associated with deterioration in glycemic control that starts during Ramadan and extends for at least one month afterwards. Attention should be directed to hyperglycemia during Ramadan and the month after Ramadan as well. A particular attention should be made to the underestimation of insulin requirements for Iftar and Suhoor meals and this may vary based on risk profile and history of overindulgent behavior during Ramadan.

## Data Availability

The raw data supporting the conclusions of this article will be made available by the authors, without undue reservation.
