# Supplementary material for: Altered Ramadan fasting glycemic profiles of adults with type 1 diabetes reveal strong evidence of underestimated insulin adjustments: a 3-year observational study in Arab settings
Source: Front Endocrinol (Lausanne). 2025 May 29;16:1399990. doi: 10.3389/fendo.2025.1399990 (PMC12158741; doi:10.3389/fendo.2025.1399990)
Supplement: Supplementary file 1 [file Table1.docx]

Supplementary Material

# Supplementary Table

**Table S1**: Average glucose levels of T1D participants before, during and after Ramadan overtime versus Controls.

| **Time** | **T1DM (N=39)** | | | | | | | | | **Controls**  **(N=49)** |
| --- | --- | --- | --- | --- | --- | --- | --- | --- | --- | --- |
|  | **2020** | | | **2021** | | | **2022** | | |  |
|  | ***Before*** | ***During*** | ***After*** | ***Before*** | ***During*** | ***After*** | ***Before*** | ***During*** | ***After*** |  |
| **0:00-2:00** | 173.5 ± 26.2 | 179.5 ± 24.6 | 160.4 ± 25.5 | 174.1 ± 26.6 | 171.0 ± 28.7 | 172.6 ± 27.2 | 172.4 ± 25.6 | 176.5 ± 29.4 | 180.2 ± 27.6 | 101.8 ± 10.3 |
| **2:00-4:00** | 177.7 ± 26.9 | 187.3 ± 24.5 | 169.2 ± 24.3 | 172.1 ± 26.7 | 176.3 ± 28.5 | 173.5 ± 29.0 | 166.5 ± 29.2 | 179.7 ± 29.2 | 178.8 ± 25.8 | 106.8 ± 11.8 |
| **4:00-6:00** | 173.4 ± 25.9 | 187.3 ± 25.0 | 169.2 ± 21.6 | 166.4 ± 27.9 | 185.4 ± 28.8 | 173.5 ± 30.4 | 162.9 ± 27.4 | 191.8 ± 27.6 | 176.6 ± 26.4 | 105.5 ± 12.6 |
| **6:00-8:00** | 165.1 ± 24.6 | 186.3 ± 24.7 | 169.2 ± 22.4 | 160.0 ± 28.4 | 178.3 ± 27.1 | 166.4 ± 29.0 | 161.5 ± 26.4 | 187.1 ± 27.9 | 173.4 ± 26.9 | 97.4 ± 9.9 |
| **8:00-10:00** | 158.7 ± 23.0 | 172.9 ± 24.2 | 161.1 ± 24.3 | 158.7 ± 27.2 | 167.5 ± 26.8 | 166.2 ± 26.8 | 162.0 ± 27.1 | 182.9 ± 27.8 | 172.6 ± 27.8 | 94.9 ± 8.9 |
| **10:00-12:00** | 157.9 ± 23.1 | 154.8 ± 25.4 | 163.8 ± 23.3 | 157.9 ± 27.8 | 158.9 ± 26.8 | 164.6 ± 25.5 | 161.8 ± 27.7 | 177.2 ± 28.0 | 168.7 ± 28.3 | 94.6 ± 9.2 |
| **12:00-14:00** | 159.6 ± 23.7 | 149.9 ± 23.9 | 158.7 ± 24.3 | 156.7 ± 28.1 | 156.0 ± 26.4 | 161.6 ± 25.3 | 161.6 ± 28.4 | 172.9 ± 28.7 | 168.3 ± 28.5 | 91.8 ± 9.0 |
| **14:00-16:00** | 155.6 ± 24.4 | 156.9 ± 22.1 | 158.3 ± 23.0 | 164.6 ± 24.5 | 159.9 ± 27.1 | 165.5 ± 25.4 | 165.5 ± 27.6 | 168.0 ± 28.7 | 170.8 ± 26.8 | 88.2 ± 8.4 |
| **16:00-18:00** | 159.3 ± 25.4 | 156.9 ± 22.3 | 168.5 ± 22.6 | 172.2 ± 27.5 | 179.2 ± 27.8 | 173.5 ± 26.5 | 164.5 ± 27.5 | 167.2 ± 28.7 | 169.6 ± 26.2 | 85.8 ± 7.8 |
| **18:00-20:00** | 170.3 ± 25.9 | 174.8 ± 24.8 | 166.4 ± 27.6 | 172.2 ± 27.5 | 179.2 ± 27.8 | 173.5 ± 26.5 | 170.3 ± 27.9 | 186.4 ± 28.5 | 176.6 ± 26.5 | 103.6 ± 10.7 |
| **20:00-22:00** | 180.2 ± 25.0 | 183.3 ± 23.6 | 171.0 ± 26.4 | 171.4 ± 30.4 | 180.8 ± 31.9 | 176.6 ± 27.3 | 173.4 ± 28.5 | 192.2 ± 27.3 | 180.5 ± 27.0 | 101.8 ± 10.3 |
| **22:00-24:00** | 180.7 ± 25.1 | 175.1 ± 25.3 | 169.9 ± 27.0 | 170.7 ± 28.7 | 176.3 ± 30.3 | 179.2 ± 28.8 | 176.7 ± 28.6 | 184.7 ± 28.3 | 180.4 ± 28.8 | 99.6 ± 10.0 |

Note: Data presented as mean ± standard deviation

**Table S2**. CGM Metrics

| CGM | **2020** | | | | **2021** | | | | **2022** | | | |
| --- | --- | --- | --- | --- | --- | --- | --- | --- | --- | --- | --- | --- |
|  | *Before* | *During* | *After* | *p* | *Before* | *During* | *After* | *p* | *Before* | *During* | *After* | *p* |
| TAR1 | 13.7 ± 2.3 | 14.2 ± 2.4 | 12.6 ± 2.2 | 0.51 | 11.8 ± 1.7 | 13.0 ± 1.8 | 13.1 ± 2.1 | 0.53 | 13.3 ± 1.9 | 17.7 ± 2.2*! | 15.7 ± 2.3 | 0.008 |
| TAR2 | 24.9 ± 1.7 | 26.1 ± 1.6 | 24.8 ± 1.9 | 0.49 | 26.4 ± 1.6 | 28.2 ± 1.5 | 26.6 ± 1.6 | 0.20 | 24.6 ± 1.3 | 27.9 ± 1.3 | 26.3 ± 1.5 | 0.007 |
| TIR | 54.9 ± 3.0 | 54.0 ± 3.0 | 55.7 ± 3.0 | 0.55 | 56.6 ± 2.5 | 53.9 ± 2.4 | 55.4 ± 2.8 | 0.21 | 56.6 ± 2.6 | 50.6 ± 2.7 | 53.4 ± 2.9 | 0.001 |
| TBR1 | 4.0 ± 0.4 | 3.5 ± 0.5 | 4.1 ± 0.5 | 0.44 | 4.0 ± 0.6 | 3.9 ± 0.5 | 4.0 ± 0.5 | 0.98 | 4.5 ± 0.5 | 3.2 ± 0.5 | 3.6 ± 0.6 | <0.001 |
| TBR2 | 2.4 ± 0.5 | 2.1 ± 0.8 | 2.8 ± 0.8 | 0.68 | 1.2 ± 0.5 | 0.9 ± 0.3 | 0.8 ± 0.2* | 0.48 | 1.0 ± 0.2* | 0.6 ± 0.2* | 0.9 ± 0.2* | 0.02 |

**Note**: Data presented as mean ± SE; ‘*’ denotes significance compared to 2020 of the same timeframes; ‘!’denotes significance to 2021 of the same timeframes; significance at <0.05.
